# Surgical Anatomy

**Published:** 1851-01

**Authors:** 


*Surgical Anatomy.* By JOSEPH MACLISE, Surgeon, with colored plates. Philadelphia: LEA & BLANCHARD. 1850.

We called the attention of our readers some time since to this excellent work, now in course of publication. The three first volumes are already issued, and but one volume remains to be published. It is, without doubt, the best work upon Surgical Anatomy in this country. No plates that we have ever seen, will compare with these in point of accuracy or clearness. The price for this complete work is eight dollars.

For sale by G. H. Derby, & Co. F. H. H.

